# The hepatocellular carcinoma risk in patients with HBV-related cirrhosis: a competing risk nomogram based on a 4-year retrospective cohort study

**DOI:** 10.3389/fonc.2024.1398968

**Published:** 2024-05-16

**Authors:** Dandan Guo, Jianjun Li, Peng Zhao, Tingting Mei, Kang Li, Yonghong Zhang

**Affiliations:** ^1^ Interventional Therapy Center for Oncology, Beijing You’An Hospital, Capital Medical University, Beijing, China; ^2^ Biomedical Information Center, Beijing You’An Hospital, Capital Medical University, Beijing, China; ^3^ Beijing Research Center for Respiratory Infectious Diseases, Beijing, China

**Keywords:** hepatocellular carcinoma (HCC), competing risk, multiple imputation, prediction, HBV-related cirrhosis

## Abstract

**Objective:**

The study aimed to build and validate a competitive risk nomogram to predict the cumulative incidence of hepatocellular carcinoma (HCC) for patients with hepatitis B virus (HBV)-related cirrhosis.

**Methods:**

A total of 1401 HBV-related cirrhosis patients were retrospectively enrolled from January 1, 2011 to December 31, 2014. Application of 20 times imputation dealt with missing data using multiple imputation by chained equations (MICE). The patients were randomly divided into a training set (*n* = 1017) and a validation set (*n* = 384) at a ratio of 3:1. A prediction study was carried out using a competing risk model, where the event of interest was HCC and the competing events were death and liver transplantation, and subdistribution hazard ratios (sHRs) with 95% CIs were reported. The multivariate competing risk model was constructed and validated.

**Results:**

There was a negligible difference between the original database and the 20 imputed datasets. At the end of follow-up, the median follow-up time was 69.9 months (interquartile range: 43.8–86.6). There were 31.5% (442/1401) of the patients who developed HCC, with a 5-year cumulative incidence of 22.9 (95%CI, 20.8%–25.2%). The univariate and multivariate competing risk regression and construction of the nomogram were performed in 20 imputed training datasets. Age, sex, antiviral therapy history, hepatitis B e antigen, alcohol drinking history, and alpha-fetoprotein levels were included in the nomogram. The area under receiver operating characteristic curve values at 12, 24, 36, 60, and 96 months were 0.68, 0.69, 0.70, 0.68, and 0.80, and the Brier scores were 0.30, 0.25, 0.23, 0.21, and 0.20 in the validation set. According to the cumulative incidence function, the nomogram effectively screened out high-risk HCC patients from low-risk patients in the presence of competing events (Fine–Gray test *p* < 0.001).

**Conclusion:**

The competitive risk nomogram was allowed to be used for predicting HCC risk in individual patients with liver cirrhosis, taking into account both the association between risk factors and HCC and the modifying effect of competition events on this association.

## Introduction

Hepatocellular carcinoma (HCC) accounts for 85%–90% of primary liver cancer, making it the fourth most common and second deadliest cancer in China (1). Hepatitis virus infection, alcohol consumption, non-alcoholic steatohepatitis, and older age mainly lead to liver cirrhosis, which is the main risk factor of HCC (2). Most hepatitis B virus (HBV)-induced HCC patients have a background of cirrhosis in China (3). HBV infection accounts for 63.9% of cancer deaths and cases in China (4).

The current guidelines recommend a monitoring interval of 6 months (3, 5, 6) for patients with liver cirrhosis. Widely available monitoring tests include tumor markers such as alpha fetoprotein (AFP) as well as various imaging techniques including ultrasound (US), computed tomography (CT), and abdominal magnetic resonance imaging (MRI). Clinical cohort studies support a biannual HCC monitoring strategy based on ultrasound (US), which improves the clinical outcomes at a reasonable cost (7, 8). Compared to annual CT, the combination of AFP and biannual US monitoring is more sensitive in detecting HCC (9). However, the advantages of the US strongly depended on the quality of the equipment and the professional knowledge of ultrasonic instruments (10). It was more cost-effective of a clinical scoring system to screen high-HCC-risk patients with cirrhosis before the diagnostic performance of US.

There is no clinical application of the HCC scoring system only applying for patients with HBV-related cirrhosis, which comprised a huge Chinese population. Currently, many models have been reported to predict HCC risk based on different etiologies. Toronto HCC risk index (THRI) scoring system (10) and our previous research (11) were applied to assess HCC risk in patients with all-cause cirrhosis. The AASL (age, albumin, sex, and liver cirrhosis)-HCC scoring system (12), real-world risk score for hepatocellular carcinoma (RWS-HCC) (13), and Chinese University (CU)-HCC score (14) were used for the prediction of HCC risk in CHB patients, taking cirrhosis into account. However, the risk of HCC varied among patients with cirrhosis of different etiologies. It is somewhat limited that these models were applied for patients with HBV-related cirrhosis (15). We are committed to develop a HCC predictive model to provide better choices for this group of patients with HBV-related cirrhosis. Moreover, from the perspective of statistical methods, these models were established using Cox proportional risk regression and Kaplan–Meier (KM) survival curve analysis and overestimated the cumulative risk of HCC (16). KM survival curves may not capture the event of interest following the occurrence of a competing event.

Liver cirrhosis is a multistate disease model, and the mortality rate increases as the disease progresses (16). Moreover, death before HCC is non-negligible, and it should always be considered a competing risk to correctly assess the HCC risks. Herein, using a large clinical cohort of HBV-related cirrhosis patients (*n* = 1401) with long-term follow-up (median, 69.9 months), we aimed to assess the HCC cumulative incidence in the presence of competing events [cirrhosis-related death and liver transplantation (LT)]. We established and internally validated a competitive risk scoring system based on Fine and Gray regression to accurately predict up-to-10-year HCC risk among patients with HBV cirrhosis.

## Materials and methods

### Patient selection

A total of 1,401 patients with HBV-related cirrhosis who were admitted at Capital Medical University, Beijing You’An Hospital, from January 1, 2011 to December 31, 2014 were included. Patients with cirrhosis were diagnosed through imaging and histological examination based on the etiology, medical history, clinical manifestations, and complications. According to the diagnosis time of liver cirrhosis, 1,401 patients were randomly divided into a training dataset (*n* = 1,017) and a validation dataset (*n* = 384) at a ratio of 3:1. We collected demographic and baseline clinical pathological information from all patients with cirrhosis, as shown below: age, sex, medical history, blood routine examination, liver and kidney function test, coagulation markers, alpha fetoprotein (AFP), and HBV viral DNA load as described in our previous study (11).

**Figure 1:**
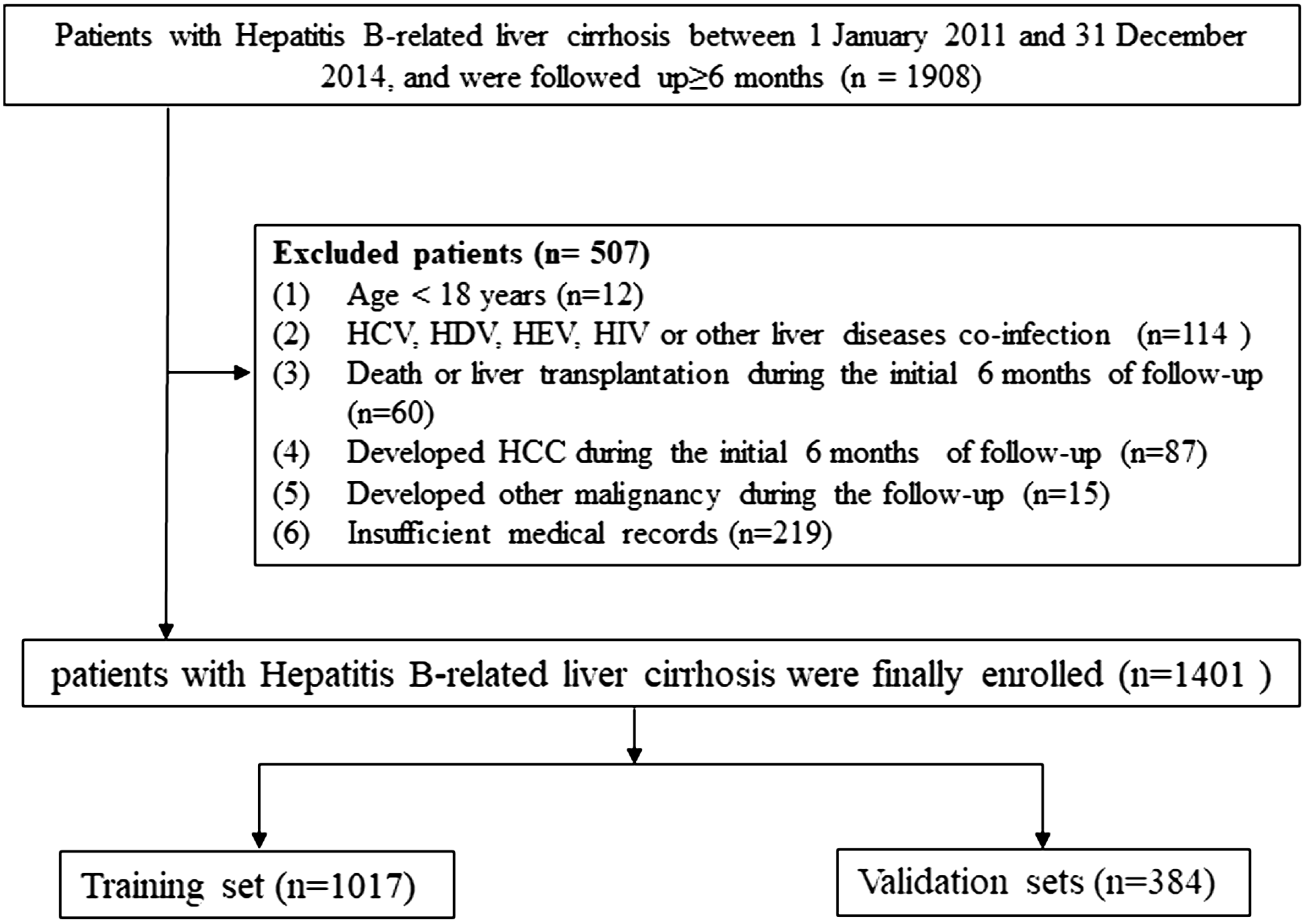
Flow chart of the enrollment in this study.

The standard of diagnosis for cirrhosis was based on Chinese guidelines on the management of liver cirrhosis (17), and for HCC it was based on the Chinese standard for the diagnosis and treatment of primary liver cancer (18). In order to minimize inter-etiological confounding of cirrhosis, the highest known risk of HCC development was set as etiological feature according to the standard of THRI methods (10). For the purpose of this study’s analysis, patients with cirrhosis who had both chronic hepatitis B and a history of alcohol or non-alcoholic steatohepatitis were classified as chronic hepatitis B (10, 19). The inclusion criteria and the exclusion criteria were described in detail in our previous study (11), and the screening process for all patients is shown in **Figure 1**.

### Outcomes and follow-up period

The enrolled patients were followed up at the outpatient clinic every 6 months, including medical examinations, laboratory tests, and ultrasound examinations (11). We calculated the follow-up since the date of cirrhosis diagnosis to the date of event occurrence (including HCC diagnosis, HBV cirrhosis-related death, and liver transplantation) or January 1, 2020, whichever occurred first. In this study, the HBV cirrhosis-related death and LT (shown by event 2) would hinder HCC (shown by event 1). Events 1 and 2 can be considered as competing events one for the other.

### Statistical analyses

Missing data could increase bias and reduce the statistical power, and application of Multiple Imputation by Chained Equations (MICE) for 20 times could reduce this impact (20). Briefly, a simple imputation was first created, and each missing value was replaced with a mean value as a “place holder”. Then, the “place holder” mean imputations of the first variable were set back to missing and then replaced with predictions (imputations) from the regression model when the first variable was the dependent variable and the other variables were independent variables. Fitting models was based on the distribution of variables, logistic regression for binary variables, linear regression for continuous variables, and Poisson model for count variables. These steps of 25 iterations for each variable that had missing values would be repeated 20 times until convergence in this study. Finally, the observed values and the 20 sets of imputed values would then constitute 20 “complete” datasets. Rubin’s rules were used to pool parameter estimates, including mean deviation, regression coefficients, standard error, derive confidence intervals, and *p*-values. Multivariate imputation by MICE to handle missing values could reduce bias in the feature selection process.

Continuous variables were represented as mean ± standard deviation or median (interquartile range, IQR). The cutoff value of quantitative variables was selected by applying surv_cutpoint function as implemented in “survminer” package. The proportional subdistribution hazard ratios (sHR) were estimated by the Fine and Gray model (21). Univariate and multivariate competing risks regression analysis were performed to select risk factors with *p*-value <0.05 for constructing the final nomogram. The cumulative incidence function curve (CIF) with Fine and Gray’s test was applied to evaluate the cumulative risk of primary outcome and competing risk events between the groups. A key assumption of CIF is that only one event can occur each time, and the subsequent occurrence of other event types are precluded. The cumulative incidence function for the *k*th cause is defined as CIFk(*t*) = Pr(*T ≤ t, D = k*), which allowed for calculating the respective CIF of events of interest and competing risk events.

The nomogram predicted the 20, 40, 60, 80, and 100 months of HCC probability among cirrhosis patients. Discrimination and predictive accuracy were assessed using the area under the time-dependent receiver operator characteristic (ROC) curve (time-dependent AUC). The consistency was evaluated using a calibration curve with Brier scores and Harrell’s concordance index. Basing on the established model, we predicted high-risk and low-risk groups with HCC cumulative incidence rate. CIF analysis and Fine and Gray’s test were used to compare the cumulative incidence rate curves of the two groups. R (version 4.2.2) software was applied for all statistical testing and visual analysis. Extension packages, including “rms”, “cmprsk,” “riskRegression,” “pec”, and “timeROC,” were also used. A *p*-value <0.05 was considered statistically significant.

## Result

### Multiple imputation for missing data in baseline characteristics

A total of 1,401 liver cirrhosis patients, from January 1, 2011 to December 31, 2014, who met the eligibility criteria were retrospectively enrolled. We assessed the demographic, laboratory, and clinical characteristics between the original database and the 20-times-imputation datasets (**Table 1**). The most missing data in clinical parameters (PT, PTA, INR, fibrinogen, and thrombin time) were 130 (9.3%). The rest of the variables had a missing proportion of less than 1.57%. The negligible difference between the original database and the 20 imputed datasets allowed for the usage of the latter for predicative research of cirrhosis patients’ outcome.

**Table 1:** Characteristics comparison of participants for the original database and 20 times multiple imputation datasets.

	Original data (missing number, value)		Pooled MI datasets
Age (years, IQR)	0	50.23 (42.52–57.21)	–
Sex (male/female)	0	998/403	–
Events (alive/HCC/death and LT)	0	821/442/138	–
Ascites (none/some/much)	0	864/482/55	–
Hepatic encephalopathy (yes/no)	0	102/1,299	–
Gastrointestinal bleeding (yes/no)	0	83/1,318	–
Hepatic failure (yes/no)	0	29/1,372	–
Antiviral therapy (yes/no)	0	809/592	–
Alcohol drinking (yes/no)	0	313/1,088	–
Alanine aminotransferase (U/L)	7 (0.49%)	39 (26.0–69.0)	39 (26–68.9)
Aspartate aminotransferase (U/L)	7 (0.49%)	44 (31.75–75)	44 (31.58–74.68)
WBC count × 10^9^/L	15 (1.07%)	4.02 (2.97–5.27)	4.02 (2.97–5.27)
Neutrophil count × 10^9^/L	15 (1.07%)	2.29 (1.63–3.19)	2.29 (1.63–3.19)
Lymphocyte count × 10^9^/L	15 (1.07%)	1.17 (0.77–1.64)	1.18 (0.77–1.64)
Monocyte count × 10^9^/L	15 (1.07%)	0.26 (0.18–0.36)	0.26 (0.18–0.36)
Hemoglobin (g/L)	15 (1.07%)	128 (108–146)	128 (108–146)
Platelet count × 10^9^/L	15 (1.07%)	76.0 (52–112)	76.1 (52–112)
Total bilirubin (μmol/L)	7 (0.49%)	24.2 (16.4–38.5)	24.20 (16.42–38.30)
Direct bilirubin (μmol/L)	7 (0.49%)	5.2 (3.4–10.53)	5.2 (3.4–10.50)
Total protein (g/L)	7 (0.49%)	68.15 (61.58–73.3)	68.20 (61.60–73.29)
Albumin (g/L)	7 (0.49%)	37.45 (31.6–42.5)	37.49 (31.69–42.5)
Globulin (g/L)	7 (0.49%)	29.5 (25.8–33.6)	29.5 (25.8–33.6)
γ-GT (U/L)	8 (0.57%)	46 (27–87)	46 (27–87)
Alkaline phosphatase (U/L)	8 (0.57%)	87.0 (66–113)	86.7 (66–113)
Prealbumin (mg/L)	8 (0.57%)	101 (62–147)	101 (62–147)
Total bile acid (μmol/L)	8 (0.57%)	18.4 (7–43.45)	18.4 (7.05–43.51)
Cholinesterase (U/L)	8 (0.57%)	4,290 (2,794.25–6,453.5)	4,292.15 (2,799.15–6,449.33)
Cholesterol (mmol/L)	11 (0.79%)	3.61 (2.92–4.38)	3.61 (2.92–4.38)
Prothrombin time (s)	130 (9.28%)	13.9 (12.3–16)	13.6 (12.18–15.56)
Prothrombin time activity (%)	130 (9.28%)	73.2 (60.7–86.95)	76.73 (62.77–89.06)
International normalized ratio	130 (9.28%)	1.2 (1.07–1.37)	1.17 (1.06–1.35)
Fibrinogen (g/L)	130 (9.28%)	1.78 (1.37–2.25)	1.84 (1.44–2.33)
Thrombin time (s)	130 (9.28%)	19.3 (17.2–21.2)	19.28 (17.23–21.18)
HBsAg (IU)	3 (0.21%)	887.5 (336.85–1,560.5)	887.72 (337.49–1,562.15)
HBeAg (positive/negative)	3 (0.21%)	510/888	512/889
HBV DNA (positive/negative)	0	910/491	–
Alpha fetoprotein (ng/mL)	22 (1.57%)	4.69 (2.31–12.84)	4.69 (2.30–12.85)
Child–Pugh (A/B/C)	0	748/411/242	–
Family history of CHB (yes/no)	0	540/861	–
Family history of liver cancer (yes/no)	0	77/1,324	–

MI, multiple imputation; IQR, interquartile range; HBsAg, hepatitis B surface antigen; HBeAg, hepatitis Be antigen; γ-GT, gamma-glutamyltransferase.-, N.A.

### Follow−up and patient outcomes

**Figure 2:**
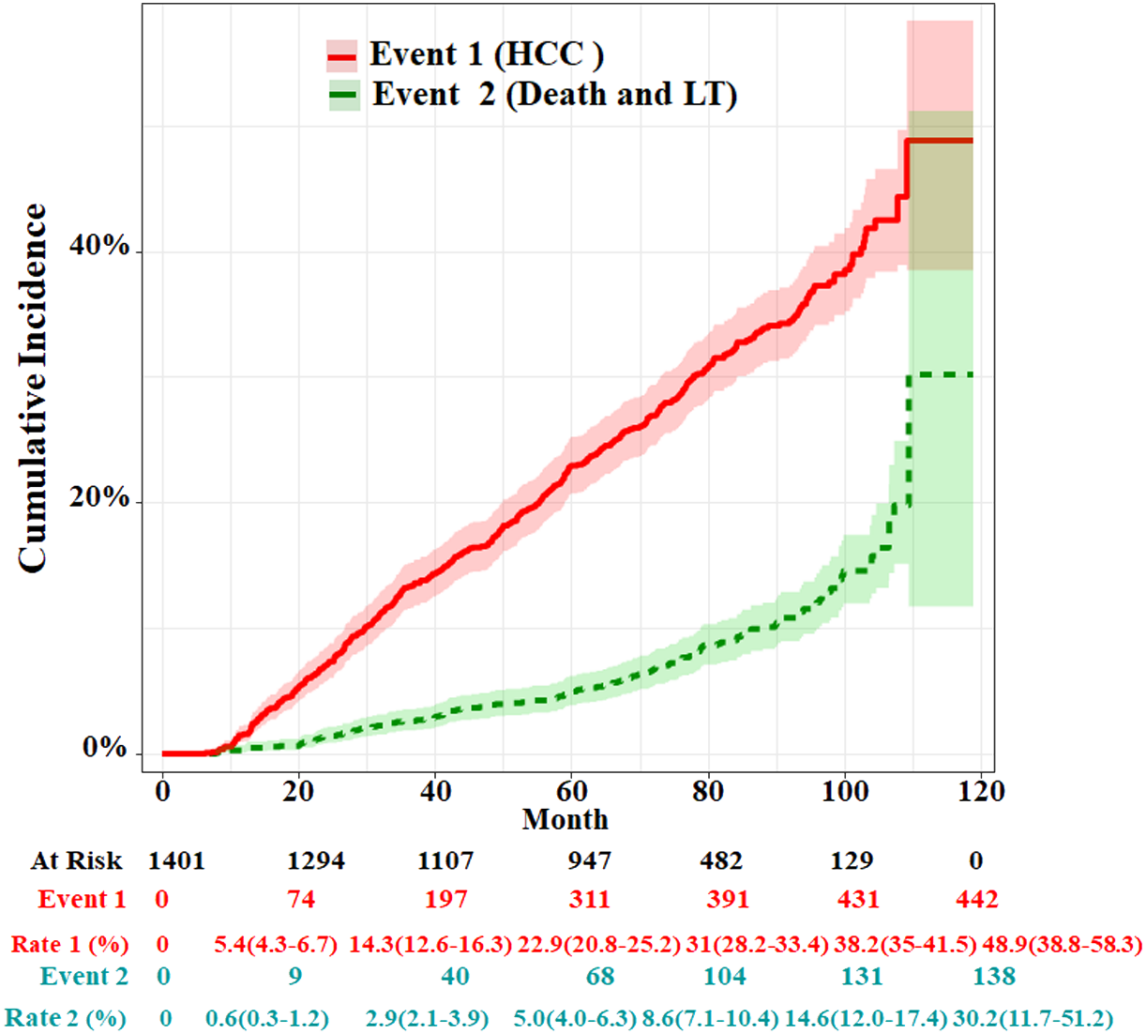
Cumulative incidence functions for HCC and competing risks event in the whole cohort. LT, liver transplantation.

The median follow-up time was 69.9 months (IQR: 43.8–86.6). By the end of the follow-up, 80 cirrhosis patients died and 58 received LT; therefore, 138 cases were set as competitive risk events (event 2). A total of 442 patients developed HCC and were set as event of interest (event 1). The cumulative HCC incidences of 1, 3, 5, and 7 years were 1.6% (95%CI, 1.1%–2.3%), 13.3% (95%CI, 11.6%–15.2%), 22.9 (95%CI, 20.8%–25.2%), and 32.2% (95%CI, 29.6%–35.0%), respectively. The cumulative incidences of death and LT at 1, 3, 5, and 7 years were 0.3% (95%CI, 0.1%–0.7%), 2.6% (95%CI, 1.9%–3.6%), 5.0 (95%CI, 4.0%–6.3%), and 9.1% (95%CI, 7.5%–10.8%), respectively (**Figure 2**). The characteristics of HCC diagnosed at the end of the follow-up are summarized in **Supplementary Table S1**. In the HCC stage, above 60% of patients had single or small tumors or BCLC stage A, and in about 83.2% of patients metastasis did not occur.

### Variable selection for predicting HCC

**Figure 3:**
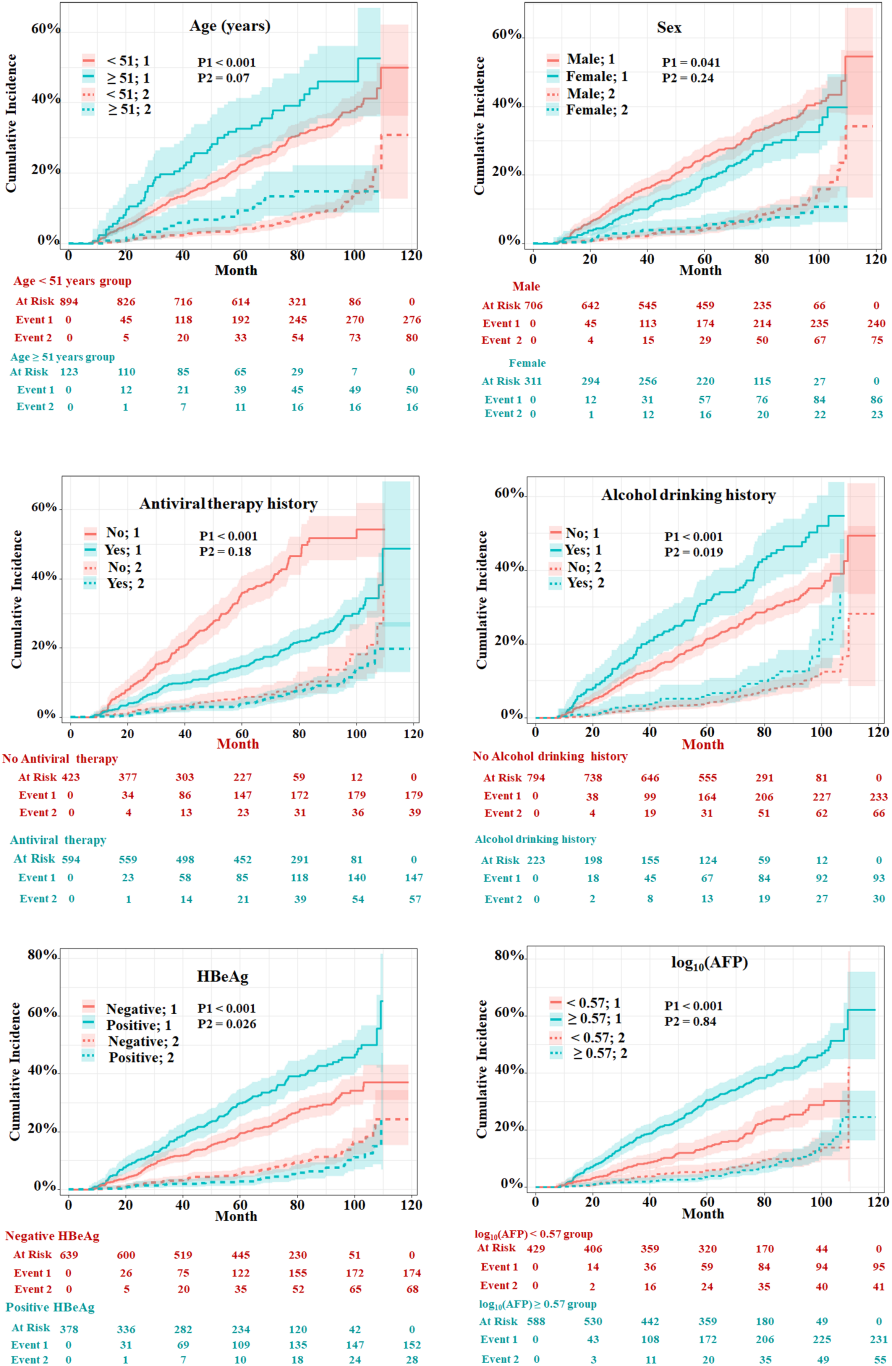
Evaluation of cumulative incidence rate for HCC of predictive risk factors in patients with HBV-related cirrhosis of the training cohort. “1” represents the outcome as HCC; “2” represents the outcome as competing risks (cirrhosis-related death and liver transplantation). The *p*-values were determined using Fine–Gray test.

The univariate and multivariate competing risk regression analyses in 20 training imputed datasets were performed to select the predicting factors of HCC and estimate the respective sHRs (**Table 2**). Univariate analysis showed that nine variables including age, sex, antiviral therapy, alcohol drinking, family history of CHB, alanine transaminase, hepatitis B e antigen (HBeAg), hepatitis B surface antigen (HBsAg), and alpha fetoprotein (AFP) were associated with the risk of HCC. After multivariate competing risk regression analysis, six independent risk factors including age, sex, antiviral therapy history, alcohol drinking history, HBeAg, and AFP were finally identified and incorporated into the model. Cumulative incidence curve analyses of the six prognostic factors were plotted based on Fine–Gray test (**Figure 3**). It could be seen that the HCC risk had a statistical increase in the male group, older age (≥51 years) group, positive of HBeAg group, unacceptance of antiviral therapy group, alcohol drinking group, and high AFP level [log_10_ (AFP) ≥ 0.57) group (all Fine–Gray test, *p* < 0.05). The sHR of the prognostic factors are outlined in **Table 2**.

**Table 2:** Univariate and multivariate Fine–Gray competing risk regression analyses in the training set (pooled MI datasets).

	Univariate analysis		Multivariate analysis	
	sHR (95%CI)	*p*-value	sHR (95%CI)	*p*-value
Age (years)	1.02 (1.01–1.03)	1.30e-07	1.04 (1.03–1.04)	7.09e-13
Sex (female vs. male)	0.77 (0.71–1.01)	0.033	0.58 (0.46–0.75)	2.31e-04
Antiviral therapy history (yes/no)	0.43 (0.36–0.52)	1.12e-13	0.46 (0.38–0.56)	7.35e-11
Alcohol drinking history	1.37 (1.16–1.64)	0.0025	1.43 (1.15–1.78)	7.30e-03
Family history of CHB (yes vs. no)	1.39 (1.16–1.67)	0.0026	1.33 (1.09–1.62)	0.175
Family history of liver cancer (yes vs. no)	1.78 (1.27–2.53)	0.057		
Alanine aminotransferase (U/L), ≥40 vs. <40	1.34 (1.14–1.58)	0.015	1.09 (0.89–1.34)	0.48
Aspartate aminotransferase (U/L), ≥40 vs. <40	1.18 (0.99–1.39)	0.11		
Total bilirubin (μmol/L), ≥50.8 vs. <50.8	0.71 (0.57–0.89)	0.097		
Direct bilirubin (μmol/L), ≥10.7 vs. <10.7	0.78 (0.65–0.95)	0.33		
HBeAg (positive/negative)	1.69 (1.44–1.98)	5.95e-08	1.52 (1.24–1.86)	7.78e-04
log_10_ (HBsAg), (IU)	1.29 (1.16–1.44)	1.51e-04	0.99 (0.88–1.12)	0.98
Total protein (g/L), ≥65 vs. <65	1.07 (0.91–1.25)	0.50		
Albumin (g/L), ≥40 vs. <40	1.89 (0.71–5.02)	0.28		
γ-GT (U/L), ≥50 vs. <50	1.00 (0.99–1.01)	0.128		
Alkaline phosphatase (U/L), ≥125 vs. <125	1.14 (0.96–1.38)	0.21		
Hemoglobin (g/L), ≥130 vs. <130	1.31 (1.12–1.54)	0.051		
International normalized ratio	0.85 (1.65–1.13)	0.37		
Fibrinogen (g/L)	1.11 (0.99–1.233)	0.13		
MLR, ≥0.44 vs. <0.44	0.84 (0.70–1.00)	0.112		
NLR, ≥1.56 vs. <1.56	0.85 (0.71–1.03)	0.18		
PLR, ≥53.5 vs. <53.5	0.83 (0.69–1.00)	0.109		
log_10_ (AFP), (ng/mL)	1.47 (1.30–1.65)	1.5e-07	1.49 (1.27–1.55)	1.44e-05

sHR, subdistribution hazard ratios; MI, multiple imputation; IQR, interquartile range; HBsAg, hepatitis B surface antigen; HBeAg, hepatitis Be antigen; γ-GT, gamma-glutamyltransferase; MLR, monocyte-to-lymphocyte ratio; NLR, neutrophil-to-lymphocyte ratio; PLR, platelet-to-lymphocyte ratio; AFP, alpha fetoprotein.

### Establishment and internal validation of the nomogram

**Figure 4:**
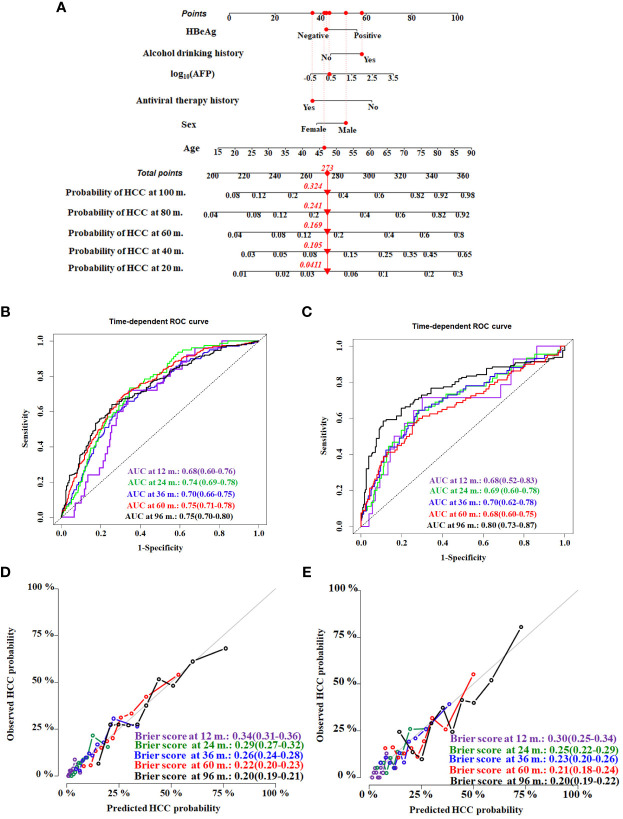
Construction and validation of the HCC competing risk nomogram for predicting the probability in HBV-related cirrhosis patients. **(A)** HCC competing risk nomogram. Time-dependent ROC curves by nomogram for HCC occurrence probability at 12, 24, 36, 60, and 96 months in the training cohort **(B)** and the validation cohort **(C)**. Calibration curves of nomogram in terms of agreement between predicted and actual HCC occurrence probability at 12, 24, 36, 60, and 96 months in the training cohort **(D)** and the validation cohort **(E)**. AUC, area under receiver operating characteristic curve.

The HCC competing risk nomogram was established in 20 imputed training datasets based on the following six independent predictive factors: age, sex (female or male), antiviral therapy history (yes or no), HBeAg (positive or negative), alcohol drinking history (yes or no), and log_10_ (AFP). The coefficients of competing risk nomogram are shown in **Supplementary Table S2**. This model could be used to calculate the probability of HCC occurrence for each cirrhosis patient—for example, a 46.36-year-old and alcohol-drinking male cirrhosis patient with 2.56 ng/mL of AFP, accepting antiviral therapy and HBeAg negative at diagnosis of cirrhosis, had a total score of about 273, and the respective 20-, 40-,60-,80-, and 100-month HCC incidences were about 4.1%, 10.5%, 16.9%, 24.1%, and 32.4% (**Figure 4A**).

Evaluating model overfitting was performed through bootstrap internal validation method. After 1,000 bootstrap cross-validation iterations, the adjusted C-index of the model was 0.75 (95%CI, 0.71–0.79). The time-dependent AUC was used to validate the discriminative ability of the nomogram. The time-dependent AUC values for the prediction of HCC at 12, 24, 36, 60, and 96 months in the training cohort were 0.68 (95%CI, 0.60–0.76), 0.74 (95%CI, 0.69–0.78), 0.70 (95%CI, 0.66–0.75), 0.75 (95%CI, 0.71–0.78), and 0.75 (95%CI, 0.70–0.80), respectively (**Figure 4B**). The adjusted Brier scores of the calibration curve for the model at 12, 24, 36, 60, and 96 months were 0.34 (95%CI, 0.31–0.36), 0.29 (95%CI, 0.27–0.32), 0.26 (95%CI, 0.24–0.28), 0.22 (95%CI, 0.20–0.23), and 0.20 (95%CI, 0.19–0.21) (**Figure 4C**), respectively. Similarly, the time-dependent AUC values were assessed in the validation cohort at 12, 24, 36, 60, and 96 months, which were 0.68 (95%CI, 0.52–0.83), 0.69 (95%CI, 0.60–0.78), 0.70 (95%CI, 0.62–0.78), 0.68 (95%CI, 0.60–0.75), and 0.80 (95%CI, 0.73–0.87), respectively (**Figure 4D**), and the adjusted Brier scores were 0.30 (95%CI, 0.25–0.34), 0.25 (95%CI, 0.22–0.29), 0.23 (95%CI, 0.20–0.26), 0.21 (95%CI, 0.18–0.24), and 0.20 (95%CI, 0.19–0.22) (**Figure 4E**).

### Performance of the competitive risk nomogram

**Figure 5:**
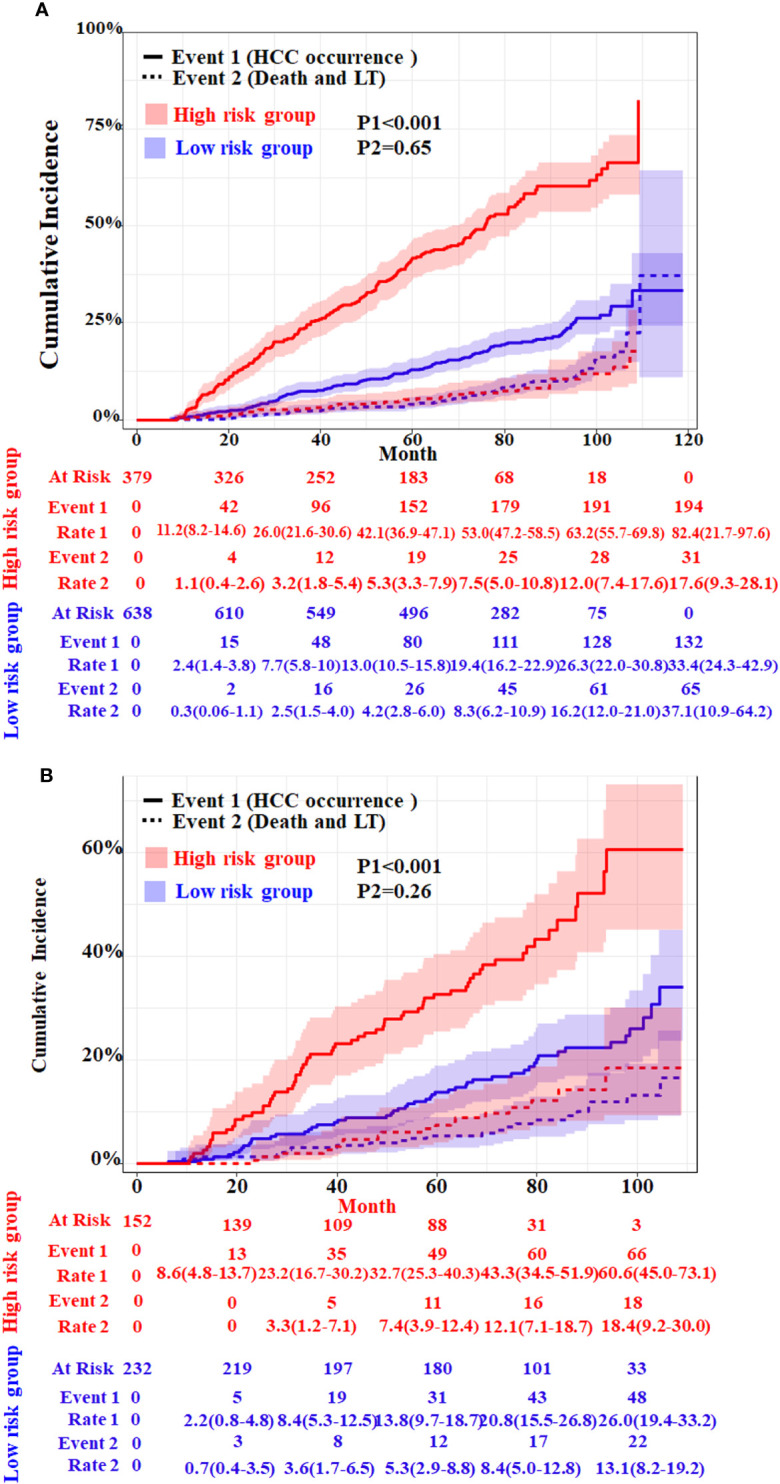
Cumulative incidence with 95%CI of HCC and competing risks event in the low- and high-risk groups of HBV-related cirrhosis patients in the training cohort **(A)** and the validation cohort **(B)**. LT, liver transplantation. The *p*-values were determined using Fine–Gray test.

In order to further evaluate the discriminative ability of the HCC competitive risk prediction nomogram, the risk score of each cirrhosis patient was calculated. The low-risk group (score <1.67) and high-risk group (score ≥1.67) were created based on the cutoff value of the risk score, which was selected by applying surv_cutpoint function implemented in “survminer” software. Patients in the training and validation cohorts were stratified based on their risk scores of HCC in the presence of competing events. The cumulative incidence curves of HCC and competitive risk event in the two groups were drawn (**Figure 5**). The respective incidences had significant differences in the low-risk and high-risk groups both in the two cohorts (*p* < 0.001). For the training cohort, the cumulative 20-, 40- 60-, 80-, and 100-month incidences of HCC were 11.2 (95%CI, 8.2–13.6), 26.0 (95%CI, 21.6–30.6), 42.1 (95%CI, 36.9–47.1), 53.0 (95%CI, 47.2–58.5), and 63.2 (95%CI, 55.7–69.8) in the high-risk group and 2.4 (95%CI, 1.4–3.8), 7.7 (95%CI, 5.8–10.0), 13.0 (95%CI, 10.5–15.8), 19.4 (95%CI, 16.2–22.90), and 26.3 (95%CI, 22.0–30.8) in the low-risk group (*p* < 0.001) (**Figure 5A**). The cumulative 20-, 40- 60-, 80-, and 100-month incidences of HCC were 8.6 (95%CI, 4.8–13.7), 23.2 (95%CI, 16.7–30.62), 32.7 (95%CI, 25.3–40.3), 43.3 (95%CI, 34.5–51.9), and 60.6 (95%CI, 45.0–73.1) in the high-risk group and 2.2 (95%CI, 0.8–4.8), 8.4 (95%CI, 5.3–12.5), 13.8 (95%CI, 9.7–18.7), 20.8 (95%CI, 15.5–26.8), and 26.0 (95%CI, 19.4–33.2) in the validation cohort (**Figure 5B**). In addition, patients with a higher HCC risk did not have a higher risk of death and LT.

**Figure 6:**
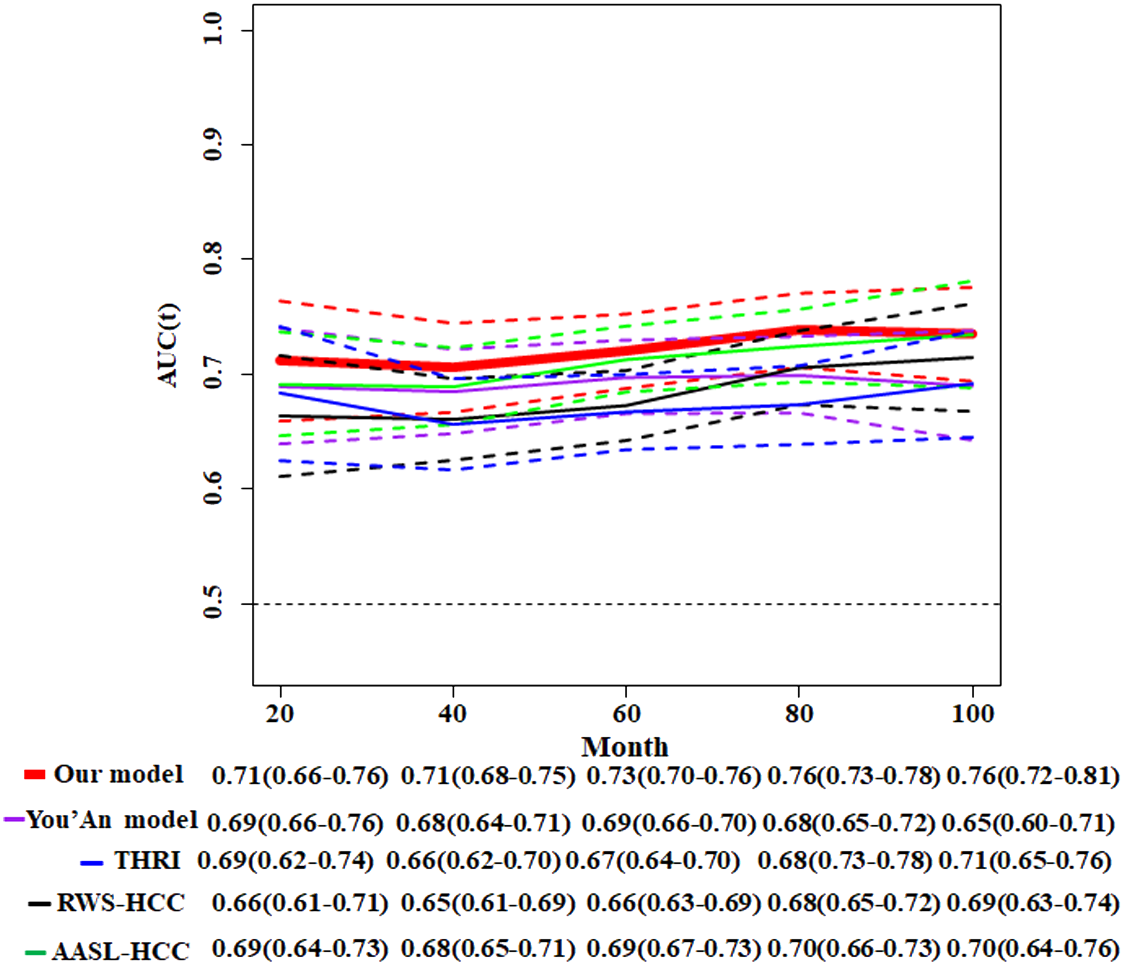
Comparison of time-dependent AUCs with 95%CIs for the competitive risk nomogram and other four HCC models. THRI, Toronto HCC risk index scoring system; You’An model, our previous research (11); AASL-HCC, age, albumin, sex and liver cirrhosis-HCC scoring system; RWS-HCC, real-world risk score for hepatocellular carcinoma.

Meanwhile, we compare our model with four other existing risk scores whose parameters all included HBV infection and cirrhosis. Toronto HCC risk index (THRI) scoring system, our previous You’an model (11), the AASL (age, albumin, sex and liver cirrhosis)-HCC scoring system, and real-world risk score for hepatocellular carcinoma (RWS-HCC) were allowed to apply our data. The result of time-dependent AUC of our model and other four models showed that our model has best discriminatory power (**Figure 6**).

## Discussion

Early screening of HCC is strongly recommended for HCC surveillance in high-risk HBV cirrhosis patients. The individualized risk of HCC varies with different etiologies of cirrhosis. In this study, we conducted a long-term follow-up (median, 69.9 months) of a large clinical cohort of patients with HBV-related cirrhosis and provided important data on the incidence rate of HCC. The establishment and validation of a competing risk model to predict the 10-year cumulative incidence of HCC in patients with HBV-related cirrhosis were pursued. During the follow-up of 0–10 years, the cumulative incidence rate of HCC in the high-risk group was significantly higher than that in the low-risk group.

The fact that the etiology of liver cirrhosis is a key determinant of HCC risk (10) indicates that there are specific risk factors for HCC in patients with HBV-related cirrhosis. After adjusting for other risk factors, the relative risk of HCC for HBsAg-positive patients alone was 9.6 (95%CI, 6.0–15.2 compared to negative patients, while the relative risk of HCC for HBsAg and HBeAg-positive patients was 60.2 (95%CI, 35.5–102.1). Positive HBeAg usually indicated active replication of HBV in hepatocytes and was an increased risk factor for HCC in CHB patients (22). In fact, liver cirrhosis patients who clear HBeAg and inhibit HBV DNA could significantly reduce the risk of HCC (23). In this study, positive HBeAg is also an increased risk factor for HCC among cirrhosis patients. It is currently clear that antiviral therapy reduces the HCC risk in CHB patients with or without cirrhosis.

Liver cirrhosis is a typical multistate model of disease progression (24); its clinical states mostly include compensated and decompensated cirrhosis and advanced decompensated state (16). The mortality rate varies in different states. In untreated patients with decompensated state, death occur in approximately 30% in 1 to 2 years after the index bleeding. Ascites is associated with a 5-year mortality of about 50% in decompensated patients (25). Overt hepatic encephalopathy and/or jaundice are associated with a 5-year survival of about 20% in advanced cirrhosis (26, 27). Renal function impairment (28), liver dysfunction, and bacterial infections (29) are associated with organ failures and high mortality in advanced cirrhosis. Competing events (cirrhosis related-death and LT) are frequent in liver cirrhosis. Death should always be considered a competing risk for assessing the incidence of HCC event in the course of the disease. If a competing event is treated as considered data, the probability of an event is overestimated using the Kaplan–Meier method (30–33). Competing risk analysis is based on the CIF to predict the probability of any event occurring first, resulting in a desirable total probability from zero to one (or the sum of probabilities for each event) (16). Meantime, because of the occurrence of competing events precluding the occurrence of event of interest, its probability does not necessarily approach unity in the end (34).

In this study, we applied Fine–Gray models and CIF to assess the risk factor and cumulative incidence of HCC in the presence of competing risks. The risk factors, i.e., alcohol drinking (yes or no) and HBeAg (positive or negative) at diagnosis of cirrhosis, were significantly correlated with HCC (both *p* < 0.001). Meanwhile, they also were slightly associated with competing events (both *p* < 0.05). The other four predictive factors, log_10_(AFP), age, sex (female or male), and antiviral therapy (yes or no), were all significantly associated with HCC (both *p* < 0.05). However, they did not show an association with competing events (both *p* > 0.05). The cumulative risk incidence of HCC and competing events were both evaluated simultaneously using these variables. Meanwhile, our model was allowed to be used for predicting HCC risk in individual patients with liver cirrhosis, taking into account both the association between risk factors and HCC and the modifying effect of competition events on this association.

This study also had limitations. Firstly, due to the retrospective nature, selection bias is inevitable, and further external validation is needed to increase the extrapolation of the model. Secondly, risk factors from common laboratory tests in hospitals were fully analyzed in this study. Transaldolase and aldolase B regulated the reprogramming of pentose phosphate pathway to have a deep effect on hepatocellular carcinogenesis (35–37). Thus, the novel metabolic markers should be comprehensively evaluated as risk variables that might improve predictive performance. Thirdly, potential interactions between risk factors need to be explored to evaluate the effects on outcomes.

## Conclusions

In the present study, we provided a systematic estimation of HCC in HBV-related liver cirrhosis patients using a retrospective cohort followed up for more than 10 years. Moreover, we established and validated a competing risk nomogram to predict the HCC risk, which might be a convenient and predictive tool for HCC screening.

## Data availability statement

The original contributions presented in the study are included in the article/**Supplementary Material**. Further inquiries can be directed to the corresponding authors.

## Ethics statement

The studies involving humans were approved by Ethics Committee of the Beijing You’An Hospital. The studies were conducted in accordance with the local legislation and institutional requirements. The ethics committee/institutional review board waived the requirement of written informed consent for participation from the participants or the participants’ legal guardians/next of kin because this article is a retrospective study. Therefore, the institutional waived the requirement to obtain distinct written informed consent from the patients.

## Author contributions

DG: Conceptualization, Data curation, Formal Analysis, Methodology, Supervision, Writing – original draft, Writing – review & editing. JL: Conceptualization, Data curation, Writing – review & editing. PZ: Conceptualization, Data curation, Writing – review & editing. TM: Formal Analysis, Methodology, Writing – review & editing. KL: Conceptualization, Writing – review & editing. YZ: Conceptualization, Data curation, Project administration, Writing – review & editing.
