# Supplementary material for: The hepatocellular carcinoma risk in patients with HBV-related cirrhosis: a competing risk nomogram based on a 4-year retrospective cohort study
Source: Front Oncol. 2024 May 16;14:1398968. doi: 10.3389/fonc.2024.1398968 (PMC11137271; doi:10.3389/fonc.2024.1398968)
Supplement: Supplementary file 1 [file Table_1.docx]

**Supplementary table 1**. Characteristics of hepatocellular carcinoma at diagnosis (n=442).

| **Variables** | **Number** | **Percentage (%)** |
| --- | --- | --- |
| Age (years, IQR) | 53.8(47.3-59.7) |  |
| Sex (Female/Male) | 115/327 |  |
| Tumor number (n) |  |  |
| 1 | 302 | 68.3% |
| 2 | 40 | 9.1% |
| ≥3 | 100 | 22.6% |
| Tumor size (mm) |  |  |
| ≤30 | 275 | 62.2% |
| 31-50 | 94 | 21.2% |
| ≥51 | 73 | 16.6% |
| Tumor stage (BCLC) |  |  |
| 0 | 5 | 1.1% |
| A | 301 | 68.1% |
| B | 58 | 13.1% |
| C | 57 | 12.9% |
| D | 21 | 4.8% |
| Metastasis (n) |  |  |
| Not | 368 | 83.2% |
| Haematogenous metastasis | 43 | 9.8% |
| Distant metastasis | 31 | 7.0% |

Note: IQR: interquartile range

| **Supplementary table 2**. Coefficients of competing risk nomogram. | | | | |
| --- | --- | --- | --- | --- |
|  | β | sHR (95% CI) | S.E. | p Value |
| Age (years) | 0.03794755 | 1.039(1.028-1.05) | 0.00548 | 4.5e-12 |
| Sex (Female vs Male) | -0.32319876 | 0.724(0.554-0.945) | 0.13603 | 1.8e-02 |
| Antiviral therapy history (yes vs no) | -0.66789199 | 0.513(0.412-0.638) | 0.11112 | 1.8e-09 |
| Alpha-fetoprotein (AFP)  (ng/ml), log_10_ | 0.23150466 | 1.26(1.066-1.49) | 0.08536 | 6.7e-03 |
| Alcohol drinking history (yes vs no) | 0.35045394 | 1.42(1.114-1.810) | 0.12389 | 4.7e-03 |
| HBeAg (positive vs negative) | 0.34178321 | 1.407(1.126-1.759) | 0.11377 | 2.7e-03 |

Note: S.E. = standard error; sHR = sub-distribution hazard ratio; CI = confidence interval.
